# Online 3D Displacement Measurement Using Speckle Interferometer with a Single Illumination-Detection Path

**DOI:** 10.3390/s18061923

**Published:** 2018-06-13

**Authors:** Min Lu, Shengjia Wang, Laura Bilgeri, Xian Song, Martin Jakobi, Alexander W. Koch

**Affiliations:** Institute for Measurement Systems and Sensor Technology, Technical University of Munich, Theresienstraße 90/N5, 80333 Munich, Germany; shengjia.wang@tum.de (S.W.); l.bilgeri@tum.de (L.B.); xian.song@tum.de (X.S.); m.jakobi@tum.de (M.J.); a.w.koch@tum.de (A.W.K.)

**Keywords:** speckle interferometry, 3D displacement, spatial carrier phase-shifting technique, nondestructive testing

## Abstract

Measurement systems for online nondestructive full-field three-dimensional (3D) displacement based on the single-shot and multiplexing techniques attract more and more interest, especially throughout the manufacturing industries. This paper proposes an accurate and easy-to-implement method based on an electronic speckle pattern interferometer (ESPI) with single illumination-detection path to realize the online nondestructive full-field 3D displacement measurement. The simple and compact optical system generates three different sensitivity vectors to enable the evaluation of the three orthogonal displacement components. By applying the spatial carrier phase-shifting technique, the desired information can be obtained in real time. The theoretical analysis and the measurement results have proven the feasibility of this ESPI system and quantified its relative measurement error.

## 1. Introduction

Due to the increased manufacturing demand for advanced materials and applications, especially in the industries of robotics, automotive, and aerospace, the technology for online measuring nondestructive full-field three-dimensional (3D) displacement is gradually becoming of great interest [1,2]. Since the 1970s, various optical approaches have been proposed. Two of the most common methods are digital image correlation (DIC) or digital speckle photography (DSP) extended through stereovision [3,4,5], and electronic speckle pattern interferometry (ESPI) with different sensitivity vectors [6,7,8,9,10]. The former techniques, which require a multi-camera system, identify the position of each object point in two frames and subsequently the relative displacement by applying a correlation algorithm to stochastic intensity patterns on the object’s surface [11,12]. The vision-based sensors with multiple cameras belong to this technique [13,14,15,16,17]. They use a full projection matrix as the transformation metric to measure the 3D structural displacement. The computer vision-based sensors have various industrial applications—for example, to control and check visual appearance in the production process, to monitor static and dynamic displacements of civil engineering structures, to assess the quantitative structural integrity, to monitor low- and high-frequency vibration of engineering structures, and to measure surface traction fields to represent the magnitude and direction of force applied to the skin’s surface, etc. However, this method can be challenging under the conditions of small depth of field or limited accessibility for detectors, etc. [18,19]. Moreover, the displacements to be measured are photographically resolved [20]. On the other hand, with the help of phase-shifting techniques in ESPI, the measurement results at nanoscale resolution can be obtained by building the relationship between the displacement and the phase change distribution caused by the interference between the object wave and reference wave [21]. To introduce different sensitivity vectors, an interferometric configuration with multiple illuminations and single detector is widely used. In this case, an online measurement can be realized by using spatial phase-shifting technique, together with, for example, the manipulation of the coherency of object and reference beams [22], or the control of the beams’ polarization [23], or the introduction of a multi-wavelength laser [24]. In addition, the ESPI systems with single illumination and multiple detectors for dynamic 3D displacement measurement have also been proposed [25,26], which, however, require a complex and elaborate calibration process. An important extension to ESPI applicability is provided by the single-shot and multiplexing measurement techniques, which, however, require complex calibration processes [27,28,29,30,31,32,33]. In year 2001, Hack et al. presented the process of validating a 3D finite element analysis by speckle interferometry using a wavelength-division multiplexing technique. It was advisable for the calibration procedure to make comparative measurements on the test object itself on selected locations [34]. Three years later, Coggrave et al. proposed a real-time interferometry system to visualize the deformation fields. A calibration process based on the minimization of a function obtained from a sequence of interferometric images was used [35]. In year 2014, Bergström et al. proposed the single-shot dual-wavelength digital holography for shape evaluation [36], whose calibration method was presented by Khodadad et al. [37,38]. In year 2015, Khaleghi et al. introduced the multiplexed holography for a single-shot three-dimensional shape and displacement measurements. A special registration algorithm is developed to minimize the differences between each two sensitivity vectors [39].

This paper proposes a new interferometric measurement system with a single illumination- detection path for online measurement of the full-field 3D displacement. In order to make the system sensible to any arbitrary displacement of the object in free space, three different sensitivity vectors are involved by a reference beam and three object beams, which are diffusely scattered from the test object and observed from different directions. By applying the single-aperture based spatial-carrier phase-shifting technique, the interferograms of the reference beam and the respective object beam in different loading events are spatially modulated and the corresponding phase distributions can be subsequently retrieved dynamically. Afterwards, with the reconstructed phase change maps, the displacement components with interferometric accuracy can be evaluated online. In addition, the performance of online measurement and the optical arrangement of this measurement system is simple, compact, and cost-effective, since neither multiple illuminations nor multiple detectors are required. Moreover, due to the single illumination-detection configuration, the calibration process referring to scaling issues is greatly simplified. Compared with the complex calibration process in the ESPI systems with multiple detectors, there is only one camera in the proposed system and the magnifications of all light paths are the same through the optical path design in advance; therefore, just one camera needs to be calibrated in the image formation process that establishes the mapping between the camera’s coordinates and the image’s coordinates. Additionally, the scales of the desired speckle interferograms generated by the interferences between the reference wave and the respective object waves do not need to be calibrated for the acquisition of the three-dimensional coordinates. Furthermore, no factors such as coherence, polarization, and spectral characteristics of the laser have to be manipulated. In this article, the working principle of this measurement system is introduced, and the experimental results obtained from a rigid-body rotation and deformation by a central load are presented and discussed.

## 2. Theory

### 2.1. Optical Arrangement

**Figure 1:**
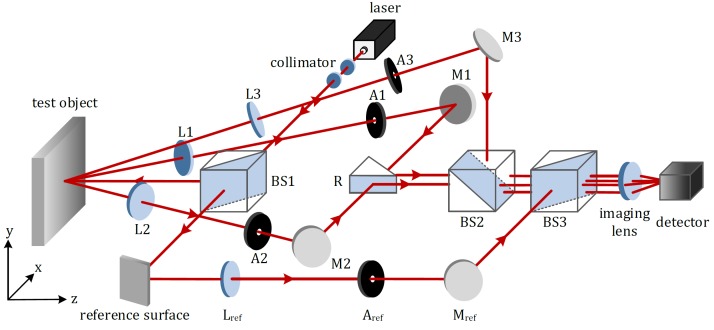
Optical arrangement of a four-beam electronic speckle pattern interferometry (ESPI) system for online 3D displacement measurement (BS: beam splitter; L: lens; A: single aperture; M: plane mirror).

The optical configuration of the proposed four-beam ESPI system for online measurement of full-field 3D displacement is illustrated in Figure 1. The expanded and collimated laser beam is firstly split into two paths by the beam splitter BS1. The reflected one illuminates the test object with optically rough surface perpendicularly, while the transmitted one strikes the reference surface. The light scattered diffusely from the test object, denoted as object beam, is observed from three different directions. Each object beam goes successively through the respective lens (L1, L2 or L3) and single aperture (A1, A2 or A3). After that, they are reflected by the plane mirrors (M1, M2 and M3), reflector *R*, and beam splitter BS2. At the same time, the light scattered from the reference surface, denoted as reference beam, passes through $L_{ref}$ and $A_{ref}$, reflected by $M_{ref}$, and meets three object beams at BS3. After travelling through the imaging lens, these four beams generate the interferometric speckle patterns at the imaging array of the detector.

It is worth mentioning that, by orienting the plane mirrors (M1, M2, M3 and $M_{ref}$) and the beam splitters (BS2 and BS3), not only the lateral displacements among the four beams on the detector, but also the relative positions of the virtual images of the single apertures (A1, A2, A3 and $A_{ref}$) in the imaging plane can be adjusted.

**Figure 2:**
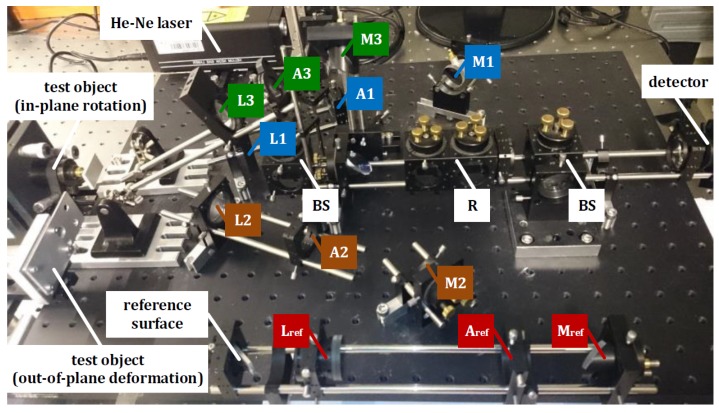
Experimental setup of online 3D displacement measurement (BS: beam splitter; L: lens; A: single aperture; M: plane mirror; R: reflector).

Figure 2 illustrates the experimental setup of the proposed interferometry system for online full-field 3D displacement.

### 2.2. Working Principle

**Figure 3:**
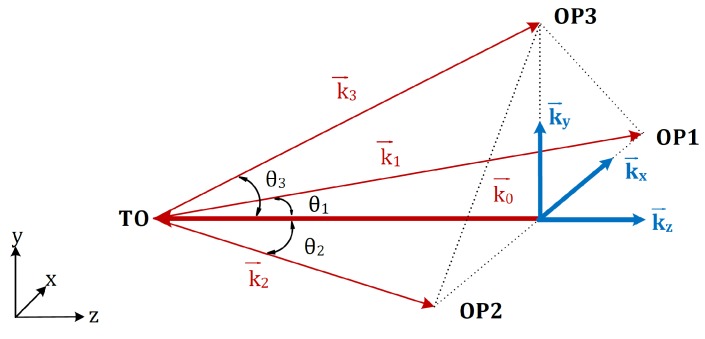
Evaluation of the sensitivity vectors (TO: test object; OP: observation point).

To describe the four-beam ESPI system clearly, the wave vectors are introduced. As shown in Figure 3, ${\vec{k}}_{0}$ denotes the wave vector of the incidence beam on the test object, while ${\vec{k}}_{1}$, ${\vec{k}}_{2}$ and ${\vec{k}}_{3}$ are wave vectors of the respective object beam. ${\vec{k}}_{0}$ lies parallel to the object’s surface normal and points along the negative *z*-axis. ${\vec{k}}_{1}$ and ${\vec{k}}_{2}$ are located in the xz-plane and are on different sides with respect to the incidence beam at angles of $\theta_{1}$ and $\theta_{2}$. ${\vec{k}}_{3}$ is in yz-plane with angle $\theta_{3}$ to the incidence beam.

Mathematically, these four wave vectors can be expressed by:

(1) $$\begin{array}{cc} \vec{k_{0}} & =\frac{2\pi }{\lambda }\left(-\vec{w}\right),\\ \vec{k_{1}} & =\frac{2\pi }{\lambda }\left(\sin\theta_{1}\cdot \vec{u}+\cos\theta_{1}\cdot \vec{w}\right),\\ \vec{k_{2}} & =\frac{2\pi }{\lambda }\left(-\sin\theta_{2}\cdot \vec{u}+\cos\theta_{2}\cdot \vec{w}\right),\\ \vec{k_{3}} & =\frac{2\pi }{\lambda }\left(\sin\theta_{3}\cdot \vec{v}+\cos\theta_{3}\cdot \vec{w}\right). \end{array}$$

Herein, λ is the wavelength of the laser; $\vec{u}$, $\vec{v}$, and $\vec{w}$ are the unit vectors along the positive xyz-coordinate axes. With these four wave vectors, an orthogonal coordinate system of the sensitivity vector $\left({\vec{k}}_{x},{\vec{k}}_{y},{\vec{k}}_{z}\right)$ can be constructed to enable the quantitative description of the object’s displacement in three dimensions.

It is to be noted that there are six interferograms generated in total, since interference occurs between each two beams $I_{i}$ and $I_{j}$ with initial phase distribution $\Delta \phi_{i}$ and $\Delta \phi_{j}$. The subscripts, i.e., *i* and *j*, are from 0 to 3, which represent the reference beam and object beams, respectively. Generally, speckle interferometer systems perform measurements by detecting the phase change $\Delta \phi_{ij}$ due to the applied load [40,41]. Thus, the intensity distribution *I* of these interference speckle patterns at a certain loading event can be expressed by:

(2) $$I=\sum_{i=0}^{3}\sum_{j=0}^{3}\left[\left(I_{i}+I_{j}\right)+2\sqrt{I_{i}I_{j}}\cos\left(\phi_{i}-\phi_{j}+\Delta \phi_{ij}\right)\right].$$

The phase changes $\Delta \phi_{ij}$ can be expressed by the well-known equation:

(3) $$\Delta \phi_{ij}=\left({\vec{k}}_{i}-{\vec{k}}_{j}\right)\cdot \vec{d},\;i=0,1,2,3;\;j=0,1,2,3,$$

with

(4) $$\vec{d}=l_{x}\vec{u}+l_{y}\vec{v}+l_{z}\vec{w}.$$

$\left({\vec{k}}_{i}-{\vec{k}}_{j}\right)$ represents the sensitivity vector, along which the displacement vector $\vec{d}$ is projected. $l_{x}$, $l_{y}$ and $l_{z}$ are three orthogonal displacement components along the respective principal axis. By substituting Equations (1) and (4) into Equation (3), the phase change distributions of six interferograms can be represented by the functions of the displacement components and the observation angles, i.e., $\Delta \phi_{ij}=f\left(l_{x},l_{y},l_{z},\theta_{1},\theta_{2},\theta_{3}\right)$. Since the observation angles are reserved as known quantities, three displacement components can theoretically be determined by solving the system of equations consisting of any three $\Delta \phi_{ij}$. Because of the geometrical relationship among the observation directions, any one of three phase change distributions of the interferograms caused by the object beams, i.e., $\Delta \phi_{12}$, $\Delta \phi_{13}$, and $\Delta \phi_{23}$, is dependent on the other two. Thus, at most two of them can be selected into the system of equations at the same time.

In this article, the example with three phase change distributions caused by the interferences between the reference beam and the respective object beam, i.e., $\Delta \phi_{01}$, $\Delta \phi_{02}$, and $\Delta \phi_{03}$, is given. By solving the equations below,

(5) $$\;\begin{array}{cc} \Delta \phi_{01} & =\frac{2\pi }{\lambda }\left[\sin\theta_{1}\cdot l_{x}+\left(\cos\theta_{1}+1\right)\cdot l_{z}\right],\\ \Delta \phi_{02} & =\frac{2\pi }{\lambda }\left[-\sin\theta_{2}\cdot l_{x}+\left(\cos\theta_{2}+1\right)\cdot l_{z}\right],\\ \Delta \phi_{03} & =\frac{2\pi }{\lambda }\left[\sin\theta_{3}\cdot l_{y}+\left(\cos\theta_{3}+1\right)\cdot l_{z}\right], \end{array}$$

three unknown displacement components $l_{x}$, $l_{y}$ and $l_{z}$ can be determined by:

(6) $$\begin{array}{cc} l_{x} & =\frac{\lambda }{2\pi }\cdot \frac{\left(\cos\theta_{2}+1\right)\Delta \phi_{01}-\left(\cos\theta_{1}+1\right)\Delta \phi_{02}}{\sin\left(\theta_{1}+\theta_{2}\right)+\sin\theta_{1}+\sin\theta_{2}},\\ l_{y} & =\frac{\lambda }{2\pi }\cdot \frac{1}{\sin\theta_{3}}\cdot \left\{\frac{\sin\theta_{1}\left[\Delta \phi_{3}-\left(\cos\theta_{3}+1\right)\Delta \phi_{2}\right]+\sin\theta_{2}\left[\Delta \phi_{3}-\left(\cos\theta_{3}+1\right)\Delta \phi_{1}\right]+\sin\left(\theta_{1}+\theta_{2}\right)\Delta \phi_{3}}{\sin\left(\theta_{1}+\theta_{2}\right)+\sin\theta_{1}+\sin\theta_{2}}\right\},\\ l_{z} & =\frac{\lambda }{2\pi }\cdot \frac{\left(\sin\theta_{2}\right)\Delta \phi_{01}+\left(\sin\theta_{1}\right)\Delta \phi_{02}}{\sin\left(\theta_{1}+\theta_{2}\right)+\sin\theta_{1}+\sin\theta_{2}}. \end{array}$$

In the case of $\theta_{1}=\theta_{2}=\theta_{3}=\theta$, the equations above can be greatly simplified:

(7) $$\begin{array}{cc} l_{x} & =\frac{\lambda }{4\pi }\cdot \frac{\Delta \phi_{01}-\Delta \phi_{02}}{\sin\theta },\\ l_{y} & =\frac{\lambda }{4\pi }\cdot \frac{2\Delta \phi_{03}-\left(\Delta \phi_{01}+\Delta \phi_{02}\right)}{\sin\theta },\\ l_{z} & =\frac{\lambda }{4\pi }\cdot \frac{\Delta \phi_{01}+\Delta \phi_{02}}{\cos\theta +1}. \end{array}$$

Furthermore, the absolute value of the synthetic displacement $|\vec{d}|$ of the test object can be obtained by the root sum square of these three displacement components by using $|\vec{d}|=\sqrt{l_{x}^{2}+l_{y}^{2}+l_{z}^{2}}$.

### 2.3. Phase Retrieval

According to the working principle, and in order to realize the online measurement of three orthogonal deformation components in an optoelectronic measurement system, three phase change distributions $\Delta \phi_{01}$, $\Delta \phi_{02}$ and $\Delta \phi_{03}$ are to be reconstructed simultaneously and dynamically. For this reason, the spatial carrier phase-shifting technique has attracted attention. By involving four single apertures, i.e., A1, A2, A3, and $A_{ref}$ from Figure 1, six different adjustable carrier frequencies can be introduced, which are able to transmit different interferograms over separate frequency areas.

**Figure 4:**
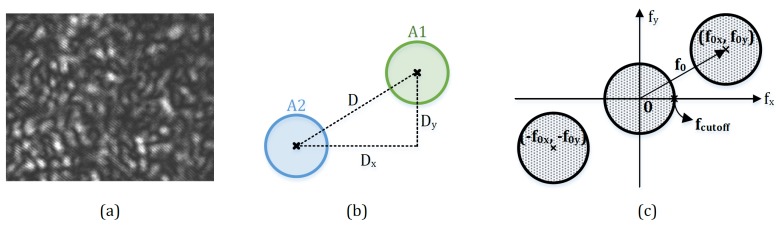
(**a**) speckle grains with internal spatial fringe structure; (**b**) relative positions of the virtual images of two single apertures in the detector’s plane; (**c**) corresponding spectrum distribution of the speckle pattern in the frequency domain.

This single-aperture based phase modulation technique generates a periodic spatial fringe structure within each speckle grain, as shown in Figure 4a, to introduce a spatial carrier frequency $f_{0}=D/2\lambda f$, where *f* represents the focus length of the imaging lens; *D* denotes the relative position and distance between the virtual images of the corresponding apertures in the detector’s plane. It is to be noted that the fringes within the speckle grains are perpendicular to the line joining the centers of two virtual apertures. Thus, by altering the orientation of the aperture pair, one can change the fringe direction over the image, and consequently adjust the direction of the carrier frequency. In addition, the aperture opening size $d_{a}$ is also an important parameter that is proportional to the spatial resolution $r_{speckle}$ and the cutoff frequency of the speckle image, i.e., $r_{speckle}=f_{cutoff}=d_{a}/2\lambda f$. Figure 4b shows an arbitrarily relative position of the virtual images of two single apertures in the plane of the detector. They are laterally displaced from each other for $D(D_{x},D_{y})$. The carrier fringes within the internally modulated speckle result in the carrier frequency $f_{0}\left(f_{0x},f_{0y}\right)$ directing along the joining line, and the carrier frequency components $f_{0x}$ and $f_{0y}$ are proportional to $D_{x}$ and $D_{y}$, respectively. The resultant spectrum of the interference speckle pattern on the detector is illustrated in Figure 4c. The spectrum pair contains the information of the interferogram, while the origin-located spectrum represents the low-frequency components of the speckle image, including the background intensity and noise. A more detailed description about this spatial carrier phase-shifting technique is demonstrated in [42].

Besides Equation (1), the interference speckle pattern *I* on the detector can be alternatively represented by the individual wavefronts [43]:

(8) $$\begin{array}{cc} I= & \sum_{i=0}^{3}\sum_{j=0}^{3}u_{i}u_{j}^{*}\;\mathrm{with}\;u=\left|u\right|e^{i\phi }\\ = & \left(u_{0}+u_{1}+u_{2}+u_{3}\right){\left(u_{0}+u_{1}+u_{2}+u_{3}\right)}^{*}\\ = & \left(u_{0}u_{0}^{*}+u_{1}u_{1}^{*}+u_{2}u_{2}^{*}+u_{3}u_{3}^{*}\right)\\ + & \left(u_{0}u_{1}^{*}+u_{1}u_{0}^{*}+u_{0}u_{2}^{*}+u_{2}u_{0}^{*}+u_{0}u_{3}^{*}+u_{3}u_{0}^{*}\right)\\ + & (u_{1}u_{2}^{*}+u_{2}u_{1}^{*}+u_{1}u_{3}^{*}+u_{3}u_{1}^{*}+u_{2}u_{3}^{*}+u_{3}u_{2}^{*}), \end{array}$$

where |u| and ϕ are the amplitude’s modulo and the phase distribution of the wave. The $u^{*}$ denotes the complex conjugate of *u*. The first four items on the right side of the equation are the self-interference components, the next six items present the cross-interference between the reference beam and object beams, while the last six items are the cross-interference components of the object beams. After applying fast Fourier transform, the intensity image is transmitted from the space domain into the frequency domain [44]:

(9) $$\begin{array}{cc} I=F(I)= & \left(U_{0}⊗U_{0}^{*}+U_{1}⊗U_{1}^{*}+U_{2}⊗U_{2}^{*}+U_{3}⊗U_{3}^{*}\right)\\ + & \left(U_{0}⊗U_{1}^{*}+U_{1}⊗U_{0}^{*}+U_{0}⊗U_{2}^{*}+U_{2}⊗U_{0}^{*}+U_{0}⊗U_{3}^{*}+U_{3}⊗U_{0}^{*}\right)\\ + & \left(U_{1}⊗U_{2}^{*}+U_{2}⊗U_{1}^{*}+U_{1}⊗U_{3}^{*}+U_{3}⊗U_{1}^{*}+U_{2}⊗U_{3}^{*}+U_{3}⊗U_{2}^{*}\right), \end{array}$$

where ⊗ denotes the convolution operation. The first four terms reside at the origin and represent the direct-current component of the image. In order to separate the other six pairs of spectra, six different carrier frequencies out of various possibilities shall be appropriately selected by fulfilling the following requirements: (1) all of the spectra must completely separate from each other; (2) all useful spectra shall be limited under the maximum spatial frequency of the detector; and (3) the cutoff frequencies are to be optimized to reach the best spatial resolution of the retrieved phase maps.

**Figure 5:**
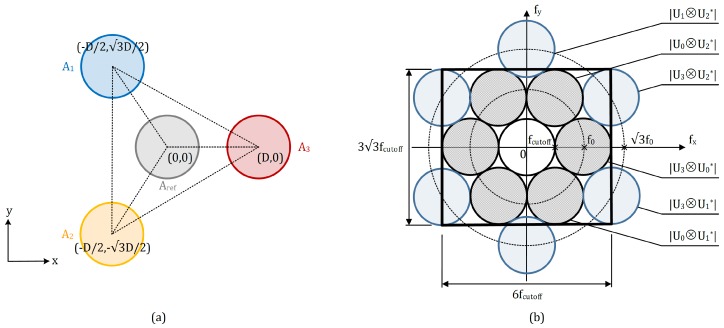
(**a**) relative positions of the virtual images of the four single apertures in the detector’s plane; (**b**) spectra distribution of the internally modulated speckle interferogram with respect to (**a**).

Based on the requirements above, the relative positions among the virtual images of the four single apertures in the system are set, as shown in Figure 5a: the virtual aperture in the reference beam $A_{ref}$ is located in the middle with coordinate (0,0); three virtual apertures $A_{1}$, $A_{2}$ and $A_{3}$ in the object beams are laterally displaced from $A_{ref}$ with the same distance *D* but at different angles of $0{}^{\circ }$, $120{}^{\circ }$ and $240{}^{\circ }$ with respect to the *x*-axis, respectively. Thus, their corresponding coordinates are $\left(-D/2,\sqrt{3}D/2\right)$, $\left(-D/2,-\sqrt{3}D/2\right)$, and (D,0). The centers of these three beams set up an equilateral triangle with side length of $\sqrt{3}D$. The resultant spectrum distribution of the internally modulated four-beam speckle interferogram is shown in Figure 5b.

The spectrum at the center represents the low-frequency components, mainly from the background intensity and noise. Supposing that the horizontal spatial carrier frequency between the reference beam and the object beam 3 is $f_{0}$, the centers of the spectra caused by the interference between the reference beam and object beams (gray-filled spectra in Figure 5b) are located on the circumference with origin (0,0) and radius $f_{0}$. On the other hand, the centers of the spectra caused by the interference between each two object beams (blue-filled spectra in Figure 5b) lie on the circumference with origin (0,0) and radius $\sqrt{3}f_{0}$. All of the spectrums are mutually tangent to each other. Additionally, the boundary of the internal seven spectra is limited directly by the spatial resolution of the detector to ensure the best spatial resolution of the retrieved phase maps because only the internal three pairs of the spectra are required for the displacement evaluation (see Equation (6)). Thereby, the cutoff frequency $f_{cutoff}$ is quantitatively half of $f_{0}$, and the spatial resolution of the detector along *x*- and *y*-axis are equal to $6f_{cutoff}$ and $3\sqrt{3}f_{cutoff}$, respectively.

After obtaining the spectra of the interferogram, the required three spectra, i.e., $|U_{1}⊗U_{0}^{*}|$, $|U_{2}⊗U_{0}^{*}|$, and $|U_{3}⊗U_{0}^{*}|$ are to be filtered out and then presented by an inverse Fourier transform to reconstruct the phase distribution of the corresponding interferogram by [8]:

(10) $$\phi_{0i}=\arctan\frac{Im(u_{i}u_{0}^{*})}{Re(u_{i}u_{0}^{*})},\;i=1,2,3.$$

Analogously, the phase distributions before and after object’s motion ($\phi_{before}$ and $\phi_{after}$) can be obtained. Then, the phase change distribution can be calculated by $\Delta \phi_{0i}=\phi_{after}-\phi_{before}$. Subsequently, the three orthogonal displacement components $l_{x}$, $l_{y}$ and $l_{z}$ can be evaluated by using Equation (6). Obviously, in the whole data processing procedures, only two interferograms under different loading events are required, which enables online measurement of the 3D displacement.

## 3. Experimental Results and Disscusion

### 3.1. Rigid-Body Rotation

In the first experiment, an aluminium plate located on a micrometer-driven rotatory stage has been examined. This stage provides three rotational degrees of freedom with $0.0140{}^{\circ }$ of rotation around the *z*-axis and $0.0095{}^{\circ }$ of tilt around *x*- and *y*-axis per revolution. The material of the reference surface is the same as the test object. Both of the surfaces are illuminated by a 0.8 mW helium-neon laser with a wavelength of 632.8 nm. To observe the interferograms, an 8-bit 1280×1024 pixels monochromatic camera with pixel pitch of 5.2×5.2 μm${}^{2}$ is used. The average size of speckles is approximately 16.0×16.0 μm${}^{2}$ that is about three times larger than the pixel pitch of the camera. The number of points for a real-time measurement is quantitatively equivalent to the resolution of the detector in use. Thus, the measurement number of points in our system is 1280×1024 = 1,310,720. On the other hand, the maximum number of points for a real-time measurement does not only depend on the resolution of the detector but also is proportional to the storage capacity and the computing capability of the system. By increasing the maximum number of points through improving either the storage capacity or the computing capability, the accuracy remains constant over time theoretically. However, the accuracy can vary by applying the detector with a higher resolution instead of the low-resolution detector because of the introduced systematic errors. The opening sizes, here referring to the diameter, of the apertures can be continuously adjusted within the range between 0.0 mm to 25.0 mm. The magnification of the measurement system is +0.89. The evaluation area of the measurement system in use is about 6×6 mm${}^{2}$. The vision area can be enlarged by decreasing the magnification of the overall imaging system, but at the expense of the decrement on the measurement resolution. The distance of the measurement system from the test object to be monitored mainly depends on the power of the laser in use. In our experimental setup (see Figure 2), the distance between the test object and lens L1 (L2) is about 135 mm, and a 0.8 mW laser is used. For longer distances, a higher-power laser is required.

**Figure 6:**
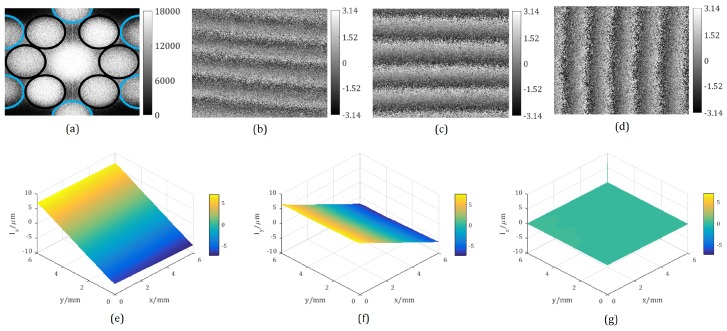
Experimental results of a rigid-body rotation: (**a**) is the spectrum of the recorded speckle interferogram; (**b**–**d**) are the wrapped phase change maps due to in-plane rotation, i.e., $\phi_{01}$, $\phi_{02}$ and $\phi_{03}$ in rad; (**e**–**g**) represent the three components of the object’s displacement, i.e., $l_{x}$, $l_{y}$ and $l_{z}$ in μm.

The test object carried out an in-plane rotation for 10 grids, equal to $0.1400{}^{\circ }$, around the *z*-axis in the clockwise direction. The measurement process was as follows: an interferogram before the object’s rotation was firstly recorded and transferred into the frequency domain by fast Fourier transform to obtain the spectrum distribution, which is shown in Figure 6a. Since the relative positions of the single apertures were set as in Figure 5a, the spectra separated from each other completely as expected. Meanwhile, only the internally-located three pairs of the spectra marked with black circles were desired for the further displacements’ evaluation. They were extracted to recover the phase maps by using Equation (10). In the same way, the phase maps after the object’s motion could also be reconstructed. Subsequently, the wrapped phase change maps $\Delta \phi_{01}$, $\Delta \phi_{02}$ and $\Delta \phi_{03}$ could be obtained, as shown in Figure 6b–d. It can be seen that the fringes from $\Delta \phi_{03}$, which relates to $l_{y}$ and $l_{z}$, inclines slightly from the vertical direction, whereas the fringes from $\Delta \phi_{01}$ and $\Delta \phi_{02}$, which are sensible to $l_{x}$ and $l_{z}$, are at tiny angles with respect to the horizontal direction. Furthermore, all the fringes are evenly spaced in the case of a rigid-body rotation [45]. Hereafter, the three wrapped phase change maps should be unwrapped and then applied for the evaluation of the three components of the displacements $l_{x}$, $l_{y}$, and $l_{z}$ by using Equation (6). The obtained displacements are displayed in Figure 6e–g. In the case of a rigid body rotation, $l_{x}\left(x,y,z\right)$ shall be the same as $l_{y}\left(y,x,z\right)$, and $l_{z}$ equals to 0. From the displacement distributions of this experiment, the ranges of $l_{x}$, $l_{y}$, and $l_{z}$ are 14.473 μm, 14.347 μm, and 0.267 μm, respectively. The relative measurement errors of $l_{x}$ and $l_{y}$ are 0.7% and 1.4%. It is evident that the out-of-plane component $l_{z}$ is two orders of magnitude smaller than the in-plane components $l_{x}$ and $l_{y}$. The reason why $l_{z}$ is not equal to 0, but much smaller than $l_{x}$ and $l_{y}$, could be the measurement errors introduced by the misaligned optical system, a miscalibrated instrument, etc.

**Figure 7:**
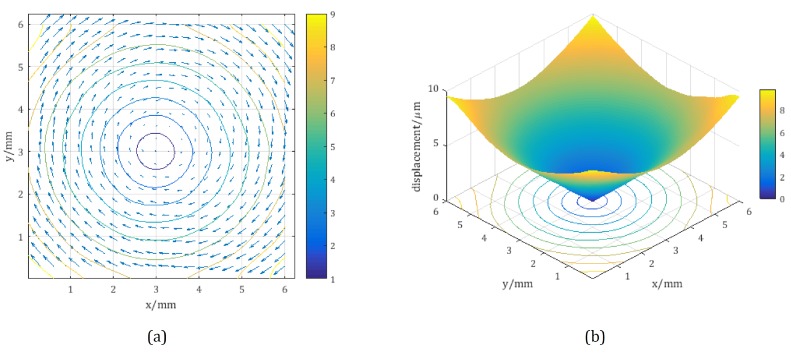
(**a**) synthetic in-plane displacements vectors of $l_{x}$ and $l_{y}$; (**b**) absolute value of the displacement |d| in μm.

The last step was to calculate the absolute value of the three-dimensional displacement caused by the in-plane rotation. As shown in Figure 7, the contours of the absolute displacement are essentially a set of concentric circles around the rotation center. The synthetic in-plane displacements vectors of $l_{x}$ and $l_{y}$ are shown in Figure 7a. The vectors are parallel to the tangential directions of the corresponding displacement contours and depict the rotation in the clockwise direction. Numerically, the absolute values of the vectors increase linearly along the radial direction.

### 3.2. Deformation by Central Load

As introduced in the above Section 2.3: “Phase Retrieval”, the single-aperture based spatial carrier phase-shifting technique is used to recover the phase distributions from the recorded speckle interferogram. By involving different adjustable carrier frequencies, three desired phase distributions can be obtained at the same time and the displacement components to be measured can subsequently be evaluated dynamically. For the purpose of verifying the performance of online measurement, an aluminium plate, which was clamped at four corners and could be continuously loaded by a micrometer head from its rear side, served as the object under investigation in the second experiment.

**Figure 8:**
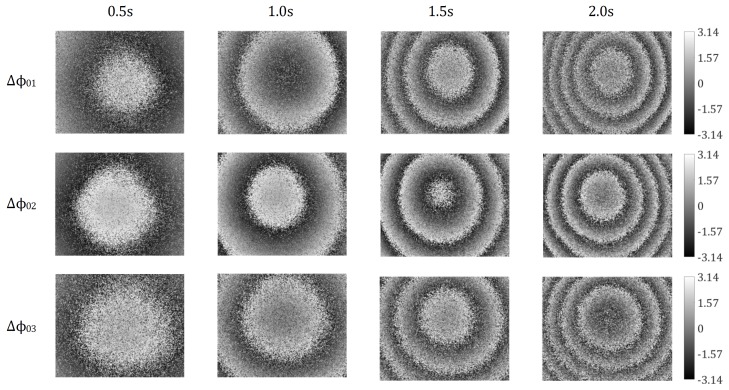
Wrapped phase change maps $\Delta \phi_{01}$, $\Delta \phi_{02}$ and $\Delta \phi_{03}$ obtained from a dynamic measurement, in which the test object was under a continuous loading process.

Figure 8 shows the array of the wrapped phase change maps obtained in a continuous loading process with the recording interval of 0.5 s.

The wrapped change maps from top to bottom represent $\Delta \phi_{01}$, $\Delta \phi_{02}$ and $\Delta \phi_{03}$ in turn, while, from left to right, they are displayed along the time-axis, i.e., in different time frames at 0.5 s, 1.0 s, 1.5 s, and 2.0 s, respectively. It is well known that, in the case of a pure out-of-plane displacement measurement, the resultant wrapped phase change maps are concentric circles around the loading point, generally the same as the measured wrapped phase change maps. This is because the out-of-plane displacement component $l_{z}$, which is contained in $\Delta \phi_{01}$, $\Delta \phi_{02}$ and $\Delta \phi_{03}$, is significantly more dominant than the other two in-plane displacement components $l_{x}$ and $l_{y}$ in this case.

The wrapped phase maps are firstly filtered by a sine-cosine average filter, and then unwrapped by either adding or subtracting the integer multiple of 2π. After that, three continuous phase change maps at each time frame have been obtained. Subsequently, Equation (6) has been applied to evaluate the corresponding principal components of displacements $l_{x}$, $l_{y}$ and $l_{z}$.

**Figure 9:**
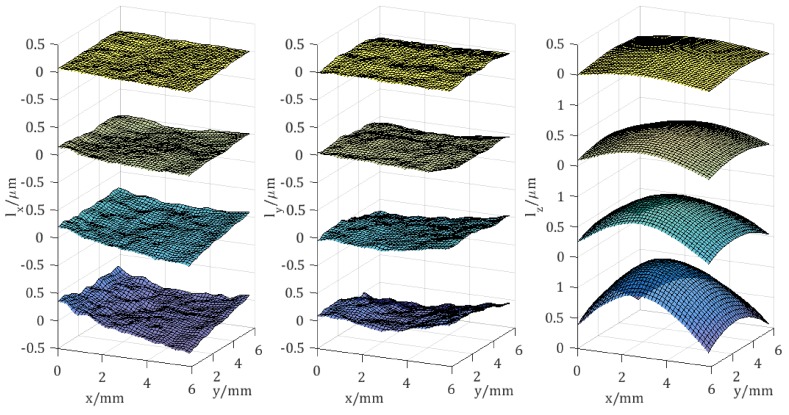
Reconstructed three orthogonal displacement components $l_{x}$, $l_{y}$ and $l_{z}$ corresponding to the time frames from top to bottom at 0.5 s, 1.0 s, 1.5 s and 2.0 s, respectively.

The results are shown in Figure 9. With the increase of the loading force, all three of the displacement components increase gradually. Compared to the simulation model of central deformation, the relative measurement error of $l_{z}$ is 0.5%. As discussed above, the out-of-plane component $l_{z}$ depicts mainly the deformed object’s surface. Since the deformation center can be observed, the in-plane components $l_{x}$ and $l_{y}$ are of opposite signs with respect to the lines, which go through the deformation center and are parallel to the *y*- and *x*-axis, respectively.

## 4. Conclusions

In this paper, a novel ESPI system with single illumination-detection path is presented for the online full-field 3D displacement measurement. To reconstruct the three orthogonal displacement components, three different sensitivity vectors are introduced by three object beams and one reference beam. The speckle patterns are internally modulated by applying the single-aperture based spatial carrier phase-shifting technique, so that the desired phase maps can be recovered from a single interferogram. After that, the phase change map and subsequently the displacement components can be reconstructed dynamically. Compared with the existing techniques, the proposed system possesses a competitive advantage: the optical arrangement is considerably simpler, more compact and cost-effective, which additionally eliminates the complicated calibration process of the imaging system and greatly simplifies the measurement operations. Therefore, this technique provides a huge variety of applications as a nondestructive testing tool in the field of aerospace, automotive, marine, and high-tech materials manufacturing.

The spatial carrier phase-shifting method applied in the proposed interferometry system increases the temporal resolution of the measurement results, but decreases the spatial resolution. In addition, the spatial resolution is also limited by the maximum spatial frequency of the camera in use. In our system, the maximum spatial resolution of the measurement results is $3.2\times 10^{4}$ m${}^{-1}$. It is also worth mentioning that the test object and the measurement system in our experiments are located on the same optical breadboard. Therefore, the whole system is susceptible to external disturbances, including the ground motion. However, in the case that the test object and the measurement system are not placed on the same test platform, the measurement results denote the sum of the displacement of the test object and the external disturbances. Thus, it is advised that the test object and the proposed interferometry system shall be installed on the same test platform.

Based on the proposed interferometric approach for 3D displacement measurement, further research could focus on the development of an extended ESPI system to measure the complete six degrees of freedom to describe the object’s movement in 3D space.
